# Improved Peripheral Intravenous Catheter Maintenance by In-Line Mechanical Pulse

**DOI:** 10.3390/bioengineering13030279

**Published:** 2026-02-27

**Authors:** Daniel T. DeArmond, Nitin A. Das, Christopher Worrell, Steven D. Dallas, Sarfraz Khan, Stewart R. Miller, John H. Calhoon

**Affiliations:** 1 Department of Cardiothoracic Surgery, University of Texas Health Science Center San Antonio, San Antonio, TX 78229, USA; 2 Department of Pathology and Laboratory Medicine, University of Texas Health Science Center San Antonio, San Antonio, TX 78229, USA; 3 Department of Accounting, University of Louisiana at Lafayette, Lafayette, LA 70503, USA; 4 Business School, Durham University, Durham DH1 3LB, UK

**Keywords:** catheterization, peripheral/adverse effects, infusions, intravenous/adverse effects, catheters, indwelling/adverse effects

## Abstract

The World Health Organization (WHO) has identified infections associated with peripheral intravenous catheters (PIVCs) as a major health burden affecting patients across countries and income status categories, meriting particular attention from care providers and researchers. As many as 70% of inpatients worldwide require PIVC placement, making it one of the most commonly performed invasive procedures in current medical practice. WHO guidelines for preventing PIVC-related infections, including bloodstream infections, focus on maintaining optimal achievable local sterility of PIVCs. The closely related complication of PIVC infiltration has attracted a great deal of research and technological focus to mitigate tissue damage due to fluid or vesicant medication delivery through infiltrated PIVCs. In this study, we report a novel approach to anti-bacterial therapy in PIVCs based on applying a low-force pulse to the fluidic system encompassing a PIVC. A 60-beats-per-minute pulse was introduced by periodic compression of the intravenous tubing upstream from the PIVC, resulting in a fluid displacement of 1–3 mm through the PIVC and into the downstream vein. In the presence of a bacterial inoculum, this pulsatility prevented bacterial growth in PIVCs, as evidenced by near-elimination of colony formation in cultured PIVC flush effluent. The introduction of pulsatility also allowed for identifying PIVC infiltration or malplacement in an ex vivo model, as infiltrated or malplaced PIVCs did not permit pulse propagation. A computationally economical digital signal processing methodology for pulse analysis was employed, providing a statistically based “two-factor authentication” of PIVC non-infiltrated status. We believe the simple intervention described in this study has the potential to reduce PIVC-associated infections and improve the early detection of PIVC infiltration, thereby improving the quality of PIVC therapy.

## 1. Introduction

Bloodstream infections and other infections associated with PIVCs have been highlighted as endemic healthcare concerns that affect patients worldwide in all income status categories [1,2,3,4]. As many as 70% of all hospital inpatients worldwide require peripheral intravenous catheter (PIVC) placement during their hospital stay, making it one of the most common invasive procedures in current medical practice [5]. Excess mortality associated with catheter-related bloodstream infections (CRBSIs) may be as high as 23% and can be associated with antibiotic-resistant bacteria that are difficult to treat [6,7]. Data suggest that interventions to reduce infection can be successful if they promote the maintenance of PIVC optimum achievable local sterility during placement, maintenance, and access [4]. The World Health Organization (WHO) has identified maintenance protocols for PIVCs as a key research gap in preventing PIVC infections [4]. Researchers have responded to this recognized unmet need through a growing body of literature and technological effort focused on the early identification of PIVC infiltration or extravasation, conditions that can be caused by or lead to PIVC-related infections and complications [8,9].

In this study, we present a novel intervention related to PIVC maintenance that introduces pulsatility into the intravenous (IV) fluid connected upstream to a PIVC at a rate that falls within normal human physiologic pulse rates and a force much smaller than normal blood pressure. We show that this pulsatility prevents bacterial growth in PIVCs in the presence of a bacterial inoculum. We demonstrate that this pulsatility is conducted through the PIVC into the downstream vein in which the PIVC is seated, such that infiltrated/extravasated or malplaced PIVCs can be distinguished from properly functioning PIVCs based on the property of successful pulse transmission. We further introduce a computationally economical digital signal processing methodology for simple pulse analysis that provides a statistically based “two-factor authentication” of PIVC non-infiltrated status.

## 2. Materials and Methods

### 2.1. IV Tubing Components and Catheters

Standard bore sterile polyvinyl chloride (PVC) IV tubing (3.0 mm internal diameter (ID), 4.0 mm external diameter (OD); Gravity Blood Set, CareFusion, San Diego, CA, USA) was used for most of the IV setup. Silicone tubing interposition segments (Becton-Dickenson, Paramus, NJ, USA, item# 2420-0007) were used to interface with the pulse generator due to greater compressibility than PVC and were connected in-line to the PVC tubing setup. Sterile 2 in length 18 Ga IV PIVCs were obtained from Nipro Corporation (Osaka, Japan; catalogue #: NIC-18Gx2). Normal saline (B Braun, Bethlehem, PA, USA; catalogue #: R5201-01) was introduced into the IV tubing as the carrier fluid for pulse delivery and de-aired. Saline was delivered with a 10 cc syringe (Becton-Dickenson, Paramus, NJ, USA, item# 302995) that remained affixed to close off the proximal end of the IV tubing and prevent ongoing delivery or leakage of fluid from the IV tubing system during pulse generation.

### 2.2. Pulse Generation

**Figure 1:**
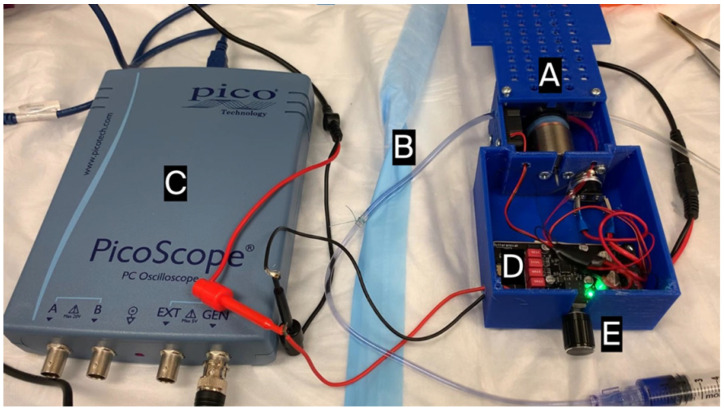
Pulse generator. (A) Voice coil motor. (B) Silicone segment of IV tubing. (C) Signal generator oscilloscope. (D) Digital amplifier. (E) Potentiometer.

A small linear voice coil electric motor (Moticont, Van Nuys, CA, USA; item #: GVCM-025-022-01) (Figure 1 (A)) was used to deliver pulses to the silicone segment of the IV tubing (Figure 1 (B)) by periodic tubing compression in short strikes against a fixed, 3D-printed surface. The motor had an outside diameter housing of 1 in, a stroke length of 0.375 in, and a maximum 4.7 N force delivery. Tubing contact was via a 1 cm diameter circular push plate. Power delivery to the linear motor resulted in tubing compression and forward fluid pulse movement in the IV tubing; pulse reversal occurred with elastic recoil of the silicone tubing upon removing power delivery. All forward fluid movement was followed by the reversal of the same volume of fluid, resulting in no net fluid propagation through the IV tubing system. Pulses or pulse patterns were programmed using the signal generator function of a PC oscilloscope (PicoScope3204B; Pico Technology, St Neots, UK) (Figure 1 (C)) and amplified with a 12 V/15 W digital amplifier (Drok, Loveland, CO, USA; product code: 2001711003) (Figure 1 (D)). Power delivery could be modulated by a potentiometer (Figure 1 (E)). Signals were programmed into the signal generator either as a fixed square wave of 1 Hz and 50% duty cycle or imported as a comma-separated value file (CSV) of 50% duty cycle square wave patterns into the arbitrary waveform functionality.

### 2.3. Bacterial Inoculum

**Figure 2:**
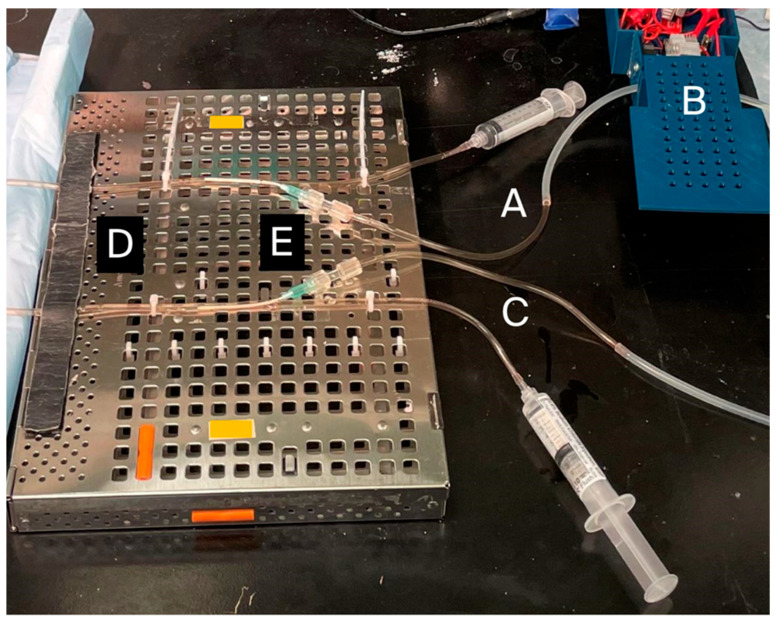
Set up for in vitro bacterial experiments. (A) Experimental arm IV tubing (receiving a pulse). (B) Pulse generator motor. (C) Control IV tubing (not receiving a pulse). (D) Tubing simulating a vein into which PIVC is inserted. (E) PIVC for experimental and control arms.

*Staphylococcal epidermidis* (*S. epi*) FDA strain PCI 1200 (ATCC, Manassas, VA, USA; item#: 12228) was sub-cultured from a −80 C Microbank bead (Pro-Lab Diagnostics, Round Rock, TX, USA; item# PL.170C/R) to a tryptic soy blood agar plate with 5% sheep blood (Hardy Diagnostics, Santa Maria, CA, USA; catalogue#: A10BX) using a four-quadrant isolation technique. The bacterial culture was grown for 18–24 h in a 35 C incubator to establish colonies. One colony was obtained as a single cell suspension in sterile saline, and 0.5 McFarland optical density inoculum was verified using a Vitek densitometer (bioMerieux, Durham, NC, USA); 100 µL of this inoculum was further diluted to a total volume of 20 mL in tryptic soy broth (Fisher Scientific, Waltham, MA, USA; catalogue #: 50-853-291) for delivery into the IV tubing apparatus. Equal volumes of tryptic soy broth inoculum were introduced into two identical IV tubing apparatuses (Figure 2) consisting of a total length of 70 cm of PVC IV tubing (as described above) with an interposed 10 cm length of silicone tubing (as described above) to facilitate pulse delivery by the pulse motor. Over 24 h at room temperature, the experimental arm IV tubing (Figure 2 (A)) received a 1 Hz pulse from the pulse generator upstream from the PIVC (Figure 2 (B)) while the control tubing (Figure 2 (C)) received no pulse. The presence or absence of active pulse delivery was confirmed by visual observation of fluid meniscus movement or lack of movement against gravity in an open IV tubing column in the IV tubing simulating the vein (Figure 2 (D)) in which the PIVC (Figure 2 (E)) was inserted. The IV tubing simulating the vein consisted of a 75 cm length of PIVC tubing as described above. All IV catheters were 18 Ga and 2 in length (as described above). After 24 h, experimental and control PIVCs (Figure 2 (E)) were removed from their tubing and flushed with 5 cc sterile saline into separate turbidity assessment test tubes. Cultures were obtained by drawing 10 µL samples from the turbidity assessment tubes for experimental and control arms using inoculation loops. Culture samples were plated on blood agar plates and maintained in a 35 C incubator for 24 h, after which colony count quantitative analysis was performed. Colony count comparison underwent a two-tailed paired t-test analysis.

### 2.4. Animals

Anesthetized Duroc swine between 50 and 70 kg were used for initial animal testing. This protocol was approved by the Institutional Animal Care and Use Committee. An 18 Ga PIVC was placed into the superficial epigastric vein, a 2–3 mm vein on the ventral surface of the pig, and directed rostrally. The pulse generator was attached to the PIVC via an IV tubing extension upstream from the PIVC, as described above, and pulses were introduced through the PIVC into the downstream vein. Doppler signals of the vein downstream of the PIVC were obtained at baseline and with pulse generation using a medical ultrasonic Doppler flow detector (IPR Medical, Tustin, CA, USA; model 811-B) or a digital spectral Doppler ultrasound system (SonoBook 8; Chison USA, Bellevue, WA, USA).

### 2.5. Photometric Velocimetry, Computer Components, Operating System, and Code

**Figure 3:**
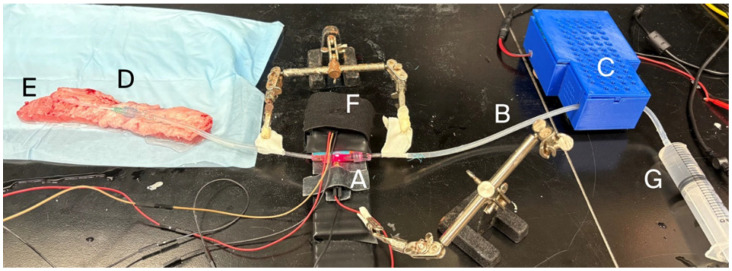
Ex vivo porcine vein photometric pulse detector set-up. (A) Photometric shutter chamber placed in-line with (B) IV tubing. (C) Pulse generator motor. (D) PIVC inserted into (E) ex vivo porcine musculo-fascial pedicle. (F) Light sensor housed in tunnel aperture. (G) Syringe saline reservoir.

Doppler ultrasound measurement of intravenous fluid pulsatility was replaced with a photometric-shutter/light break-beam mechanism to facilitate pulse signal capture in electrolytes (Figure 3). A photometric shutter chamber (Figure 3 (A)), consisting of a 3 cm length of slightly wider, clear PVC tubing of 6 mm OD and 4 mm ID (Terumo Cardiovascular, Ann Arbor, MI, USA; Item # 60053) was connected in-line with the IV tubing connected to the PIVC (Figure 3 (B)). The shutter chamber (Figure 3 (A)) was located downstream from the pulse generator (Figure 3 (C)) but upstream from the PIVC (Figure 3 (D)), which was inserted into an ex vivo musculo-fascial pedicle of porcine superficial epigastric vein (Figure 3 (E)). The shutter occupying the shutter chamber consisted of a 5 mm segment of 2 mm OD glass capillary tube colored black (Sigma Aldrich, St. Louis, MO, USA; Item#: 41121705) and was placed free floating in the chamber. Enclosing the shutter chamber tubing at both ends with Luer lock connectors prevented embolization of the floating shutter outside the shutter chamber while maintaining unobstructed continuity of the IV fluid column. Compression of the silicone IV tubing (Figure 3 (B)) by the pulse generator’s push plate (Figure 3 (C)) caused the floating shutter to be displaced forward in the shutter chamber; withdrawal of the push plate resulted in pulse flow reversal due to the elastic recoil of the silicone tubing and a return of the shutter to its original position. Back-and-forth movement of the shutter in the shutter chamber broke a light beam that passed through the shutter chamber (Figure 3 (A)), as detected by a light sensor on the opposite side of the shutter chamber from the light source (Figure 3 (F)). The light source and sensor consisted of a Receiver Sensor Module and KY-008 Transmitter Module (DEVMO corporation, Manama, BH) connected to general-purpose input/output (GPIO) pin 17 on a Raspberry Pi 4 single-board computer (Raspberry Pi Holdings plc, Cambridge, UK) running a 32-bit Raspberry Pi operating system and the Python programming language. In Python coding, a time mark was applied to light break-beam events to calculate inter-pulse intervals by subtracting the time-mark value of the previous break-beam event from the time-mark value of the current break-beam event (code in Supplementary File S1). The sensor was housed in a platform containing a tunnel aperture shielded from ambient light to minimize light scatter (Figure 3 (F)).

### 2.6. Ex Vivo Model of IV Catheter Infiltration or Malplacement

The superficial epigastric vein of anesthetized pigs undergoing euthanasia for other procedures was dissected subcutaneously and removed as an isolated vein segment of approximately 20 cm length with underlying musculo-fascial pedicle for ex vivo benchtop experiments (Figure 3 (E)). For infiltrated-vein simulation, 0.6% agar (Fisher Scientific, Pittsburgh, PA, USA; product #: BP1423500) was injected into the vein and allowed to gel to mimic thrombosis. Non-infiltrated control veins were injected with 0.6% agar in which gelation was inhibited by lowered pH (0.1 M HCl (Sigma-Aldrich, Allentown, PA, USA; product #HX0603)). Agar or gelation-inhibited agar was liquified by heating and then cooled at ambient room temperature prior to introduction into experimental or control veins, where gelation was allowed to occur for 4 h. An 18 Ga PIVC (Figure 3 (D)) de-aired with saline and connected with IV tubing to a saline reservoir (Figure 3 (G)) was placed into the mid-portion of the vein prior to agar introduction. For the PIVC malplacement simulation, the PIVC was intentionally placed in an extra-venous position in the subcutaneous tissues adjacent to the vein. Pulse generation was performed through the PIVC for all experimental trials, and pulse detection was carried out using the photometric shutter mechanism described above. Gelated and non-gelated agar trials, as well as intravenous and extravenous PIVC placements, were compared with Fisher’s exact test.

### 2.7. “Two-Factor Authentication” of IV Catheter Patency Based on Inter-Pulse Intervals

Pulse-associated light break-beam events detected by the light sensor represented a first-factor, immediate confirmation of non-infiltrated status and successful intravenous PIVC placement. A second-factor authentication system was designed in inter-pulse intervals as a safeguard against the risk of artifactual pulse detection or sensor malfunction leading to possible false negative conclusions of PIVC malfunction when relying solely on the first-factor confirmation. Sixteen-point time-domain inputs were constructed from a combination of expected and observed inter-pulse intervals in NumPy 2.4.2 (Python library) array form, with element indices numbered 0–15 (Table 1). These time-domain arrays served as inputs for fast Fourier transform (FFT) calculation in NumPy. Nine of the elements of the 16-point time-domain array, indices 0, 2, 4, 6, 7, 10, 12, 14, and 15, were defined as the “expected window” of inter-pulse intervals and populated with fixed integer time values, which did not vary between trials, with the following assignments for these indices: 1 s, 1 s, 1 s, 1 s, 2 s, 1 s, 1 s, 1 s, and 1 s, respectively (Table 1). The remaining 7 of 16 array elements, indices 1, 3, 5, 8, 9, 11, and 13, were populated with the variable, non-integer values of observed inter-pulse intervals produced by the pulse-generating apparatus in real time (Table 1). The second-factor authentication method was validated by programming five different inter-pulse interval sequence models (IISMs) into the arbitrary waveform functionality of the signal generator to produce five different patterns of observed non-integer inter-pulse interval values in the pulse-generating apparatus (Table 1). One IISM (IISM1) was the authenticating, or “true”, sequence with respect to the expected windowed values. The remaining four (IISM2–5) represented different versions of non-authenticating, or “false”, sequences that were similar but crucially different from the authenticating sequence in the time domain (Table 1). The arbitrary waveform functionality of the signal generator allows users to upload a waveform composed of any pattern of pulses, with the entire pattern repeated by the signal generator at a user-set frequency in a numeric text box in the manufacturer’s software interface. The IISMs were uploaded into the signal generator as CSV files with trains of 0 s and 1 s, which were rendered into the graphical form of a square wave sequence by the signal generator, with peak values of 1 producing power delivery to the pulse-generating motor and trough values of 0 producing power withdrawal. Each IISM was designed as a pattern of 8 square wave peaks, translating to 8 pulses delivered to the pulse motor, resulting in 7 inter-pulse intervals. The waveform peaks were constructed of square waves with evenly spaced intervals separated from adjacent peaks by the same time value, defined as 1 time unit, or, when removing a single pulse to produce a pause in the inter-pulse interval sequence, a value of 2 time units between peaks. The delivery frequency of the overall arbitrary waveform by the signal generator was set to achieve inter-pulse intervals with approximate time values at or near either 1 or 2 s for each IISM, as detailed below and in Table 1. Each of the five IISMs was trialed 52 times to produce 52 FFT datasets to ensure adequate statistical power to compare the FFT spectra produced by each IISM. The inter-pulse intervals of observed values were populated into the NumPy time-domain array in real time in the sequence in which they were generated by the light break-beam mechanism using the “insert (index, element)” coding method in Python (Supplementary File S1). When 7 inter-pulse intervals were generated, FFT calculation proceeded in real time using the len(array) method in Python to trigger an action based on the array length meeting the defined criteria for a 16 data-point FFT time domain consisting of the 7 observed inter-pulse interval values and the 9 fixed “expected” inter-pulse interval values (code provided in Supplementary File S1). Time values delivered by the pulse generator to produce the observed inter-pulse intervals for the five IISMs were, respectively, designed as follows for array indices 1, 3, 5, 8, 9, 11, and 13 (Table 1): IISM1 (authenticating sequence, paused pulse in mid-sequence): 1 s, 1 s, 1 s, 2 s, 1 s, 1 s, and 1 s; IISM2 (paused pulse earlier in the sequence): 1 s, 1 s, 2 s, 1 s, 1 s, 1 s, and 1 s; IISM3 (no pause in pulse sequence): 1 s, 1 s, 1 s, 1 s, 1 s, 1 s, and 1 s; IISM4 (higher frequency authenticating sequence): 0.9 s, 0.9 s, 0.9 s, 1.8 s, 0.9 s, 0.9 s, and 0.9 s; and IISM5 (lower frequency authenticating sequence): 1.1 s, 1.1 s, 1.1 s, 2.2 s, 1.1 s, 1.1 s, and 1.1 s. FFT was performed on the 16-point merged observed/expected dataset and graphed in real time using MatPlotLib 3.10.8 (Python graphing library) with FFT frequency bins on the *x*-axis and vector magnitudes on the *y*-axis, as per convention. Frequency values from FFT were expressed as generalized bins at 0.0625 (1/16) intervals. Of the 16 vectors returned in performing the 16-point FFT, the zero-frequency vector (vector 0) and vectors at frequencies above the Nyquist sampling limit (vectors 9–15) were discarded and excluded from analysis. Quadratic regression analysis of frequency-domain broadband curves was programmed manually in Python using the method of least squares, with regression curves and R^2^ values plotted in MatPlotLib, which was not used for statistical analysis and was only performed to aid in real-time visual assessment of the frequency-domain broadband curve’s smoothness to facilitate high-throughput data generation consistency (Supplementary File S1).

### 2.8. Statistical Analysis

An event study methodology was used to analyze the data from the IISM trials [10]. The “expected” sequence of defined inter-pulse intervals was interleaved with the “reference IISM” sequence of defined inter-pulse intervals to compose a mathematically precise “expected/reference” time-domain input (Table 1). The FFT values generated from this time-domain input, consisting of complex vector magnitudes for eight frequency bins, as described above, served as the standard broadband spectrum (Si*). The “reference IISM” values coincided with the “expected” sequence in the time domain with respect to the position in the resulting interleaved sequence of two 2 s (paused pulse) inter-pulse intervals, one 2 s interval contributed from the “expected” sequence and one from the “reference IISM” sequence, which, when paired, were immediately adjacent to each other in the final “expected/reference” time domain sequence (Table 1). Next, for each IIMS1–5 subject trial j, the observed (non-integer) sequences of inter-pulse interval values were interleaved with the “expected” sequence of inter-pulse intervals to obtain time-domain inputs that underwent FFT. We compared the resulting complex FFT vector magnitudes for each of the eight frequency bins i for each subject trial (Sj,i*) to the complex FFT vector magnitude values for the eight matching frequency bins from the “expected/reference IISM” spectrum (Si*). We summed the i deviations of the vector magnitude values for IISM1–5 relative to the “expected/reference IISM” to capture the “cumulative deviation” (CD) for each subject j, with the following equation:

$${\mathrm{CD}}_{j}\;=\;\sum_{1}^{8}\left\|S_{j,i}-S_{i}^{*}\right\|$$

for i = 1 to 8, where Sj,i was the magnitude of the ith complex FFT vector of the jth subject and Si* was the magnitude of the perfect ith complex FFT vector summed over the values for the 8 frequency bins for IISM1–5. Because the complex FFT vector magnitude values for IISM1 were designed to be as close as possible to the “reference IISM” complex FFT vector magnitude values within the standard deviation of the pulse/fluidic/optic system of pulse generation and detection, and therefore represented the “true” IISM, we performed t-test analysis for the mean CD for the IISM1 cohort versus the IISM2–5 cohorts, which were designed to be “false” IISMs. For each trial of all IISMs, the absolute value of the vector magnitude deviations was summed to obtain a CD. Differences in the CDs of IISMs2–5 versus IISM1 for all frequencies combined or for only the highest frequencies (7th and 8th frequency bins, CD_78_) underwent t-test analysis.

A subject-level analysis by group was also undertaken to identify subjects with excessive CD and establish a means for defining and identifying subjects as “true” or “false” in real time. As a basis, the study used the mean and standard deviation of the CD values of the IISM1 group to identify all subjects with excessive CD. We considered various thresholds to maximize the identification of IISM2–5 subjects with excessive CD while minimizing the number of IISM1 subjects with excessive CD as the fundamental assumption was that in a clinical scenario involving patients, the costs of accepting a subject with an IISM2–5 pulse sequence, i.e., a false negative result, would be greater than that of rejecting an IISM1 true negative pulse sequence. This analysis is tantamount to having type II errors that are more costly than type I errors. Since lower CD values are preferred and high CD values are undesirable, we created four thresholds: IISM1 mean CD + 1 standard deviation; IISM1 mean CD + 2 standard deviations; IISM1 mean CD + 3 standard deviations; and IISM1 mean CD + 4 standard deviations. This method was also applied to the cumulative deviations within the frequency magnitudes for only the highest two frequency bins (CD_78_).

## 3. Results

### 3.1. Anti-Bacterial Effect with Pulse Addition to the IV Catheter

**Figure 4:**
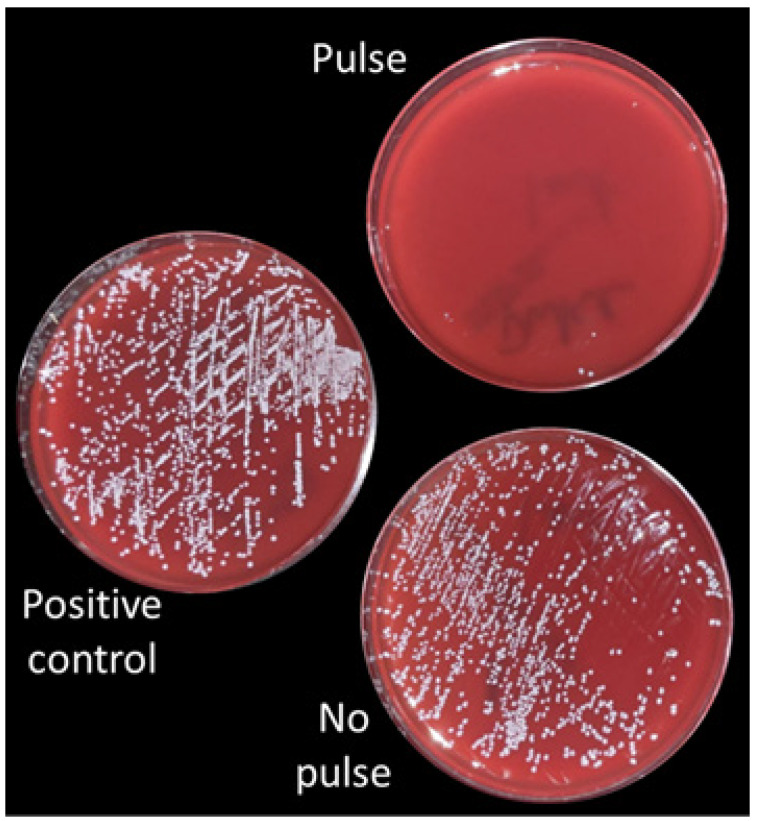
Abrogation of bacterial colony growth in cultured effluent flush from pulsed PIVC vs. control.

Adding a 1 Hz pulse to the PIVC in the presence of an *S. epidermidis* inoculum resulted in the near-complete prevention of bacterial growth in the pulsed versus non-pulsed PIVC, as demonstrated by the abrogation of colony growth from catheter flush effluent (36.3 ± 2.1 colonies in non-pulsed vs. 1.3 ± 0.6 colonies in pulsed, *n* = 3, *p* = 0.0011) (Figure 4). This was a highly reproducible result, which was more pronounced with heavier bacterial presence. The visualized pulsatile displacement of the fluid meniscus against gravity downstream of pulsed PIVCs ranged between 1 and 3 mm.

### 3.2. Ultrasound Signal of In Vivo Porcine Vein at Baseline and with Pulse Introduction

**Figure 5:**
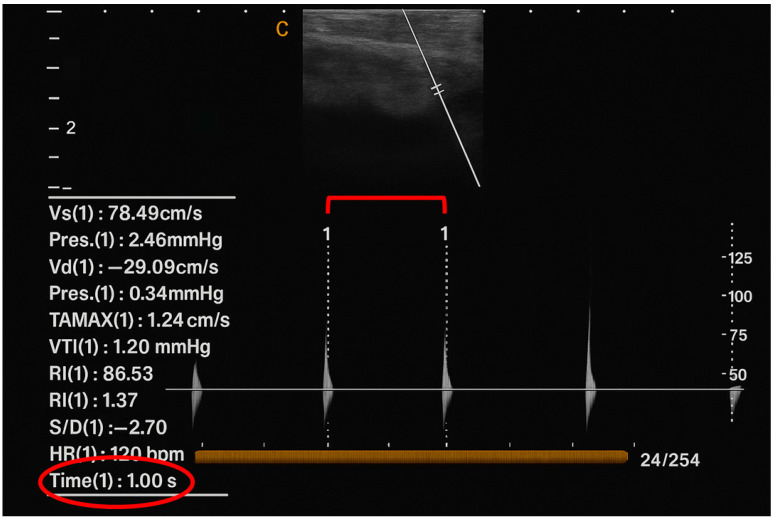
In vivo ultrasound signal of porcine vein with pulse introduction upstream of a PIVC inserted into the vein and measurements obtained downstream from the PIVC in the porcine vein. **Red bracket:** inter-pulse interval measured by the ultrasound caliper tool. **Red ellipse:** caliper-measured inter-pulse interval value read-out.

In test pigs in vivo, no Doppler signal other than minimal background noise was present at baseline in the superficial epigastric vein downstream of the IV catheter, either audibly or by spectral Doppler graphical representation, consistent with the physiologically non-pulsatile nature of mammalian peripheral veins. With exogenous pulse introduction at 1 Hz, a clearly audible 1 Hz Doppler pulse was produced. By spectral Doppler, sharp spikes were present in the graphical representation, which could be measured precisely at 1 s separation (Figure 5, red ellipse) with the caliper function of the ultrasound device software (Figure 5, red bracket). The amplitude differed slightly from pulse to pulse, but pulses were clearly distinguishable from the non-pulsatile background (Figure 5).

### 3.3. Ex Vivo Model of IV Catheter Infiltration with Shutter/Light Break-Beam Pulse Detection

The photometric shutter/light break-beam apparatus demonstrated high accuracy with pulse detection in ex vivo superficial epigastric vein pedicles, with a measured mean inter-pulse interval of 1.000 ± 0.002 s (n = 100) between break-beam events upon delivery of a 1 Hz pulse from the pulse generator. In the presence of gelated intravenous contents or IV catheter malpositioning, visual inspection of the photometric shutter showed no displacement of the shutter. No light break-beam events were registered by the light sensor (i.e., no first-factor authentication), while pulse detection was consistently retained in the presence of gelation-inhibited agar introduced into the vein (*p* = 0.0079, n = 5 for both gelated and malpositioned IV).

### 3.4. “Two-Factor Authentication” of IV Catheter Patency Based on Inter-Pulse Intervals

**Figure 6:**
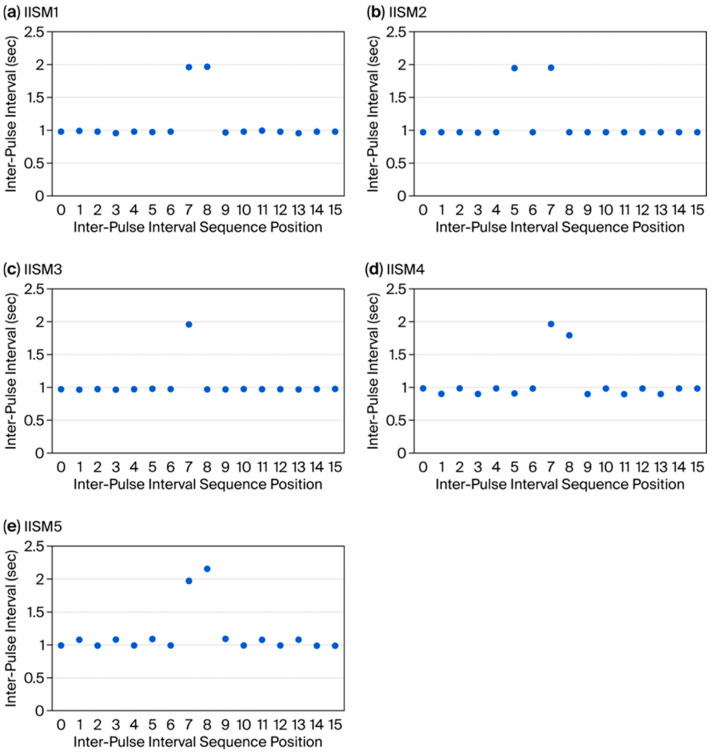
Sixteen-point time-domain graphs for inter-pulse interval sequence models (IISM1–5) interleaved with the FFT “expected window”. (**a**) Time domain array resulting from the combination of observed inter-pulse intervals of IISM1 with the “expected window”. (**b**) Time domain array resulting from the combination of observed inter-pulse intervals of IISM2 with the “expected window”. (**c**) Time domain array resulting from the combination of observed inter-pulse intervals of IISM3 with the “expected window”. (**d**) Time domain array resulting from the combination of observed inter-pulse intervals of IISM4 with the “expected window”. (**e**) Time domain array resulting from the combination of observed inter-pulse intervals of IISM5 with the “expected window”.

**Figure 7:**
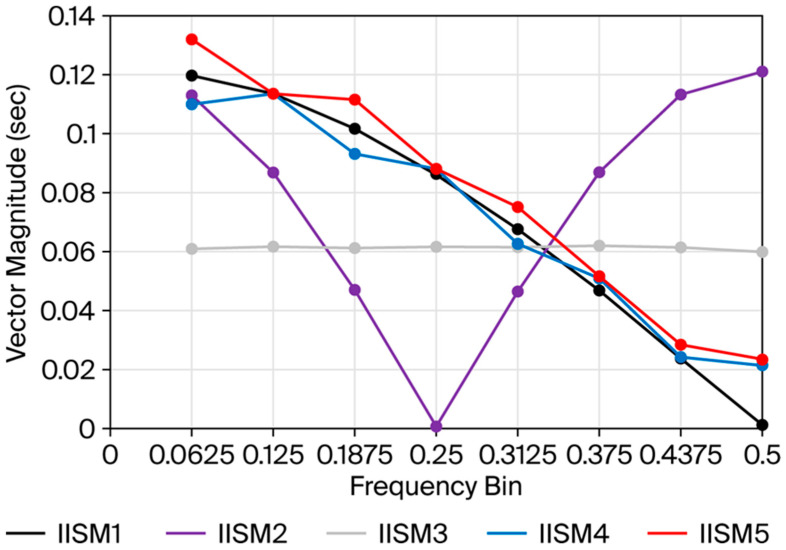
Mean broadband frequency spectra for the FFTs of the sixteen time-domain inputs constructed from sequence models IISM1–5, interleaved with the FFT “expected window” with frequency bins in generalized 0.0625 (1/16) intervals on *x*-axis and vector magnitude (sec) on *y*-axis. The vector magnitude values for the 0 frequency bin and frequency bins greater than 0.5 (alias region) were discarded and are not shown. (IISM1—black plot; IISM2—purple plot; IISM3—gray plot; IISM4—blue plot; IISM5—red plot).

**Figure 8:**
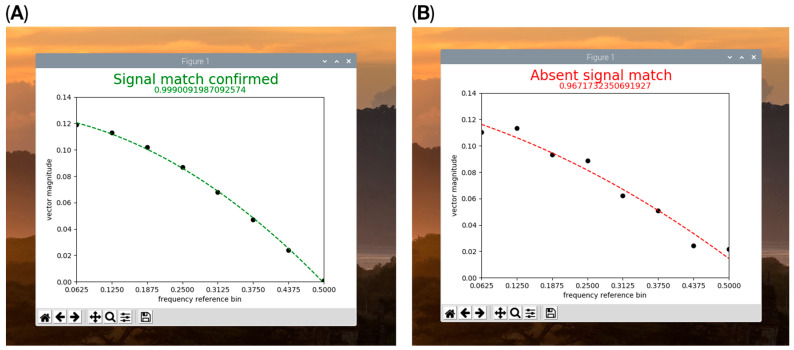
Single board computer real-time display of MatPlotLib FFT graphs for a completed trial for authenticating ((**A**), IISM1), and non-authenticating ((**B**), IISM4) inter-pulse interval trials from photometric shutter detection of pulses delivered through the PIVC apparatus in vitro. The plot in (**A**) corresponds to the plot for IISM1 (black plot from Figure 7). The plot in (**B**) corresponds to the plot for IISM4 (blue plot from Figure 7).

The time-domain array resulting from the combination of the observed inter-pulse intervals of an example trial for the authenticating sequence (IISM1) with the “expected window” values produced the approximate graphical form of a discrete rectangular (rect) function (Figure 6a). The FFT of this array resulted in a broad-band spectral pattern resembling a sinc (sin(x)/x) function consistent with the digital signal processing property of the rect and sinc functions representing a Fourier pair [11] (pp. 84–88) (Figure 7, black curve). Because the rect function was maximally compressed with respect to the time domain sampling rate, the sinc-like function demonstrated maximum expansion, with elimination of the side lobes commonly associated with the sinc function, and reduced to only the main lobe of the sinc-like function, consistent with the dilation property of the Fourier transform [11] (pp. 351–353) (Figure 7, black curve). The vector magnitudes of this spectral pattern monotonically decreased with increasing frequency, exhibiting the highest vector magnitude at the lowest frequency (Figure 7, black curve, 0.0625 frequency bin) and vector magnitude for the highest frequency near zero (Figure 7, black curve, 0.5 frequency bin). In contrast, the time-domain array resulting from the values of an example trial generated using the template of IISM2 (early pause) interposed with the “expected window” resembled two separated spikes (Figure 6b) and produced an FFT broadband spectral pattern resembling a normalized cosine (cos) function (Figure 7, purple curve), consistent with the digital signal processing property of the two delta (spike) function and cos function representing a Fourier pair [11] (pp. 90–91). The time-domain array resulting from values of an example trial for IISM3 (no pause) with the “expected window” produced the approximate graphical form of a single spike (delta) function (Figure 6c) and produced the approximate FFT broadband spectral pattern of a constant value function with approximately equal vector magnitudes at all frequencies across the broadband spectrum (Figure 7, gray curve), consistent with the digital signal processing property of a single delta (spike) function and a constant value function representing a Fourier pair [11] (p. 84). Example trials for the time-domain arrays of IISM4 and 5 with the “expected window”, similarly to the authenticating function (IISM1), produced graphical representations resembling a rect function but with greater distortion of the true rectangular curve morphology (Figure 6d,e) and FFT broadband spectral patterns more divergent from a smooth sinc-like function, particularly with consistent vector-magnitude over-expression in the highest frequency bin (Figure 7 blue curve for IISM4, red curve for IISM5, attention to 0.5 frequency bin). These differences in FFT broadband spectral distributions were readily discernible at a glance by the researcher performing data collection (Figure 7) and aided the performance of high-throughput trials. The time to complete one trial, including generating seven inter-pulse intervals in the PIVC fluidic system, calculating the FFT, and graphing the FFT magnitude curve on the computer display, was approximately 10 s per trial. The appearance on the single-board computer display of the MatPlotLib graph of an FFT is shown in Figure 8 for the authenticating (IISM1, Figure 8A) and non-authenticating (IISM4, Figure 8B) trials.

The CD of the observed IISM1 vector magnitudes from the idealized “expected/reference” vector magnitudes over all frequencies was 0.0073 s ± 0.0042. The CD for the observed vector magnitudes for IISM2–5 versus the “expected/reference” vector magnitudes all differed significantly from the CD for IISM1 (IISM2: CD = 0.4462 s ± 0.0013, *p* < 0.001 vs. IISM1; IISM3: CD = 0.2953 s ± 0.0031, *p* < 0.001 vs. IISM1; IISM4: CD = 0.0523 s ± 0.0020, *p* < 0.001 vs. IISM1; IISM5: CD = 0.0635 s ± 0.0020, *p* < 0.001 vs. IISM1) (Table 2). By setting a threshold for authentication at 3 standard deviations of the CD for the observed IISM1 sequence, a standard was established by which all trials of the observed IISM1 and none of the trials for the observed IISM2–5 met the criteria for authenticating the “true” pulse sequence based on vector magnitude CDs (Table 3). Additionally, the cumulative deviation for only the two highest-frequency bins (frequency bins 0.4375 and 0.5, the seventh and eighth frequency bins, respectively, designated as CD78) for IISM2–5 also differed significantly from that for IISM1 (IISM1 0.0017 s ± 0.0011; ISM2 0.2103 s ± 0.0008, *p* < 0.001 vs. IISM1; IISM3 0.0974 s ± 0.0013, *p* < 0.001 vs. IISM1; IISM4 0.0220 s ± 0.0012, *p* < 0.001 vs. IISM1; IISM5 0.0280 s ± 0.0010, *p* < 0.001 vs. IISM1) (Table 4). By setting a threshold for authentication at 4 standard deviations of the observed IISM1 sequence from the idealized, “expected/reference” spectrum with respect to CD78, a standard was established by which all trials of the observed IISM1 and none of the trials for the observed IISM2–5 met the criteria for authenticating the “true” pulse sequence (Table 5).

## 4. Discussion

The WHO has highlighted infections associated with the use of intravascular catheters as a major health burden affecting patients across countries and income status categories [4]. Bloodstream infections associated with PIVCs may be caused by antibiotic-resistant organisms and, as a result, may be difficult to treat [6,7]. Maintenance protocols for promoting local PIVC sterility have been identified by the WHO as a key research gap in preventing these infections [4]. The pulse delivery system employed in the current study, with its profound prevention of bacterial growth in PIVCs in the presence of a bacterial inoculum, has the potential to prevent PIVC-related bloodstream infections through improved PIVC maintenance protocols promoting local PIVC sterility. The excursion of the fluid column’s meniscus with pulse delivery at 1 Hz observed in this study was only 3 mm at most, which translated to approximately 1 mN of actual delivered force to the fluid column and thus the need for only low-power, low-cost pulse motors to generate. This small amount of fluid movement was nonetheless adequate to abrogate bacterial growth in the PIVC in an *S. epidermitidis*-inoculated in vitro model (Figure 4). The fluid velocity in the PIVC with pulse generation in this study was undoubtedly accelerated due to the principle of flow rate conservation through the smaller diameter of the PIVC with respect to the overall IV tubing diameter (approximately 0.8 mm vs. 3 mm ID), which may, in part, underlie the profound prevention of bacterial growth consistently observed in PIVCs receiving a pulse vs. stagnant PIVCs. We hypothesize that the prevention of bacterial growth in pulse-treated PIVCs could have been attributed to the prevention of bacterial quorum sensing, which relies on the accumulation of autoinducer molecules secreted by bacteria into the extracellular environment to promote high cell density growth and biofilm formation [12]. Further investigation would be required to support this hypothesis. We did not necessarily equate the abrogation of colony formation with a bactericidal effect of the introduced pulse, as the pulse was very low force, but only inhibited proliferation. We did not have a mechanism in this study to assess for bactericidal effects or biofilm formation. The strain of *S. epidermidis* chosen for this study is a non-biofilm former, but even bacterial strains that are non-biofilm formers may be triggered by quorum-sensing mechanisms to undergo proliferation toward the promotion of high cell density. Importantly, as a non-pharmacologic approach with the potential to be generalized to any bacterial strain reliant on quorum sensing, pulse maintenance therapy in PIVCs might also assist in preventing infection by antibiotic-resistant bacteria.

The ability to transmit a pulse through a PIVC differentiates functional PIVCs from infiltrated or malplaced PIVCs. The fluidic system of a PIVC inserted into the lumen of a patent vein and connected upstream to IV tubing can be considered a continuous column of liquid and, as such, obeys the property of all hydraulic systems that a force exerted in one section of the column is transmitted equally throughout the column. This property facilitated the detection of PIVC infiltration or malplacement, a condition that occurs downstream from the PIVC, by pulse detection upstream from the PIVC in the IV tubing, where accessibility is straightforward. This property was verified statistically against both infiltrated and malplaced PIVCs. Gels do not conduct a pulse because, as semi-solid materials, they do not allow for liquid volumetric displacement. Agarose gel has been used to model thrombus in vascular structures [13], and we hypothesized that infiltration is analogous to a state of thrombosis, i.e., a gel-like state. A PIVC seated in the gel-like state of an infiltrated vein effectively sees a brick wall at its distal tip with respect to pulsatile liquid displacement. Since the IV tubing upstream from a PIVC generally contains a crystalloid solution, we replaced Doppler analysis of pulse generation with a photometric device containing a floating shutter because, in early experiments we conducted, the Doppler signal in crystalloids was weaker and less consistently detectable than in blood or colloids. The need to prime the IV tubing upstream from the PIVC with a colloid to boost signal-to-noise for ultrasound monitoring would complicate the potential future regulatory approval of this device. The ability to identify an upstream pulse photometrically in the PIVC fluid column served as a “first-factor authentication” that the PIVC was properly seated and not infiltrated.

Because the device described in this study represents a diagnostic tool guiding medical decision-making with the potential to directly affect patient welfare, we felt that a “second-factor authentication” to provide enhanced certainty of PIVC functionality was indicated to rule out false negative assessments, provided it was simple to implement. False negatives could result in delivering fluids or vesicant medications into an infiltrated or malplaced PIVC based on an incorrect “first-factor” conclusion in the setting of a one-time sensor error or artifact. The second-factor authentication system, building on short sequences of slow pulses and creating an interleaved window in FFT against an “expected” inter-pulse interval pattern (Table 1), was highly computationally economical, as evidenced by the ability to execute this system using a single-board computer and rudimentary programming of a signal generator. The time to complete a trial and receive a graphical display of the FFT spectral analysis to confirm a functional PIVC was little more than the time required to generate the data, i.e., approximately 10 s. We designed the system to only minimally alter the delivered 1 Hz pulse, skipping just one pulse in an otherwise uninterrupted pulse train, since the pulse appeared to be therapeutic due to its anti-bacterial properties (Figure 4) and because this approach greatly simplified coding (Supplementary File S1). This “second-factor authentication” system, operating within the leveraged analytic power of the frequency domain of the FFT, allowed for the “true” authenticating sequence of pulses to be statistically differentiated even from sequences that were very similar in the time domain and provided a schema for identifying pulse patterns with confidence in real time based solely on differential high-frequency power expression in the broadband spectrum (Figure 7). Furthermore, it allowed for a “one-size-fits-all” analytic approach to be applied to any encountered time-domain input of inter-pulse intervals meeting the array-length selection criteria that triggered analysis as programmed into the code (Supplementary File S1). From the standpoint of ease of implementation, pulse generation, pulse detection and second-factor authentication all have the potential to be contained within a small single-board computer and can all be housed in a smart watch-style device.

The limitations of this study include the use of a free-floating shutter mechanism for photometric analysis. Due to concerns of embolism into a vein, a free-floating element in IV tubing would potentially encounter hurdles from a regulatory standpoint, but was effective in this study to establish proof of concept and could be remedied by simple tethering of the shutter to the wall of the tubing. With respect to de-airing, some patience was required to eliminate all air bubbles from the floating shutter, but this was not insurmountable, and all bubbles were meticulously eliminated from the tubing prior to initiating experimental trials. A second limitation of this study was the lack of a long-term analysis of the pulse delivery effect on the PIVC microenvironment in a peripheral vein in vivo. Pulse delivery through a PIVC as described in this study, may have deleterious effects on the vein in which the PIVC is seated, which could only be determined through longer-term observation in an in vivo setting, though we believe this is unlikely given that the pulse was a much lower force than normal human blood pressure and fell within the range of normal human heart rates. A third limitation was the choice of bacterial strain examined in this study. While coagulase-negative staphylococcus (*S. epidermidis*) is by far the most common organism cultured in peripheral and central catheter-associated bacteremia [14], the study of additional bacterial strains or, more specifically, biofilm-producing or antibiotic-resistant strains could potentially have strengthened this study. The use of agar to represent vein infiltration was also a possible limitation of this study. Agar was chosen because of the inter-relationship between peripheral vein thrombophlebitis and infiltration in the PIVC setting. The fibrin clots of thrombosis consist of proteinaceous gels and have similar physical properties to agar gels [15]. Agarose has been used to model fibrin clots in models of intravascular thrombosis [13]. However, no direct comparison between agar gel and intravenous thrombus or infiltration was undertaken in this study.

The use of FFT to analyze inter-pulse intervals in cardiology has been extensively reported [16]. The novelty in the current study was the design of specific inter-pulse intervals to generate tailored FFT spectra that were computationally economical to analyze with a very small number of observed data inputs. The approach allowed for the statistically substantiated identification of even small deviations of observed time-domain data from an expected result by simple frequency-domain, broadband spectral curve analysis that was also visually obvious to the human end user. The method of FFT described here, building in digital signal processing cardinal functions, has the potential to be applied to other fields of analysis currently limited by the need to integrate many data points with high intrinsic standard error to arrive at a rapid determination of true versus false with low computational requirements. Windowing of time-domain data in FFT is widely practiced [11] (pp. 282–292). The window employed in this study consisted of a forward model of pulses in that the sequence in the “expected window” was pre-programmed into the code with the intent to match a “true” detected pulse sequence, i.e., IISM1, within the limits of the standard deviation of pulse delivery through the PIVC fluidic system. The effect of matching sequences was to produce a sinc-like function in the frequency domain; any other detected pulse sequence deviated from that broadband pattern due to the asymmetric design of the FFT window. As a forward-model system of pulse analysis, it bears some resemblance to hypothesized neural forward models associated with the corollary discharge or efference/reafference axonal motif [17] and, as such, we suggest referring to this FFT windowing approach as an “efference window”.

## 5. Conclusions

In conclusion, introducing a low-force, physiologic-rate pulse into a PIVC has profound anti-bacterial effects in vitro, which may translate into the prevention of PIVC-associated bloodstream infections, including those due to antibiotic-resistant bacteria, in human patients. Successful pulse transmission through a PIVC confirms that the PIVC is properly seated in a vein and that the vein is not infiltrated. Because of the nature of hydraulic systems, PIVC infiltration can be reliably detected in the IV tubing upstream from a PIVC, greatly facilitating device implementation. Furthermore, pulse transmission can be analyzed using basic digital signal processing principles without substantial alteration of the form of the pulse to confirm non-infiltrated status with statistical confidence. We believe that this system could represent a low-cost, widely translatable intervention to improve care and reduce the complications associated with PIVCs.

## Figures and Tables

**Table 1:** Sixteen-point time domain inputs for the FFT “expected window”, reference IISM, and experimental IISMs1–5.

Array Position	Expected Window	Reference IISM	IISM1	IISM2	IISM3	IISM4	IISM5
0	1 s						
1		1 s	1 s ± SD	1 s ± SD	1 s ± SD	0.9 s ± SD	1.1 s ± SD
2	1 s						
3		1 s	1 s ± SD	1 s ± SD	1 s ± SD	0.9 s ± SD	1.1 s ± SD
4	1 s						
5		1 s	1 s ± SD	2 s ± SD	1 s ± SD	0.9 s ± SD	1.1 s ± SD
6	1 s						
7	2 s						
8		2 s	2 s ± SD	1 s ± SD	1 s ± SD	1.8 s ± SD	2.2 s ± SD
9		1 s	1 s ± SD	1 s ± SD	1 s ± SD	0.9 s ± SD	1.1 s ± SD
10	1 s						
11		1 s	1 s ± SD	1 s ± SD	1 s ± SD	0.9 s ± SD	1.1 s ± SD
12	1 s						
13		1 s	1 s ± SD	1 s ± SD	1 s ± SD	0.9 s ± SD	1.1 s ± SD
14	1 s						
15	1 s						

**Table 2:** All frequency bin totaled thresholds for identification of excessive cumulative deviation (CD).

Inter-Pulse Interval Sequence Model	Mean Cumulative Deviation (CD)	Standard Deviation	Min	Max	Group Comparisons t-Stat (*p* Value)
IISM1	0.0073	0.0042	0.0023	0.0193	-
IISM2	0.4462	0.0013	0.4416	0.4483	709.6 (*p* < 0.001)
IISM3	0.2953	0.0031	0.2848	0.3012	392.6 (*p* < 0.001)
IISM4	0.0523	0.0020	0.0498	0.0605	86.9 (*p* < 0.001)
IISM5	0.0635	0.0020	0.0599	0.0733	86.0 (*p* < 0.001)

**Table 3:** Subject cumulative deviation totaled over all frequency bins (CD) analysis by inter-pulse interval sequence model (IISM) groups.

	Number of Subjects Exceeding CD Thresholds		
	IISM1 CD Mean + 1 S.D.	IISM1 CD Mean + 2 S.D.	IISM1 CD Mean + 3 S.D.
IISM1	11 (21.2%)	2 (3.8%)	0 (0.0%)
IISM2	52 (100%)	52 (100%)	52 (100%)
IISM3	52 (100%)	52 (100%)	52 (100%)
IISM4	52 (100%)	52 (100%)	52 (100%)
IISM5	52 (100%)	52 (100%)	52 (100%)

**Table 4:** Only 7th and 8th frequency bin (high frequency) thresholds for identification of excessive cumulative deviation (CD).

Inter-Pulse Interval Sequence Model	Mean Cumulative Deviation Bins 7 + 8 (CD_78_)	Standard Deviation	Min	Max	Group Comparisons t-Stat (*p* Value)
IISM1	0.0017	0.0011	0.0003	0.0051	-
IISM2	0.2103	0.0008	0.2075	0.2123	1.1 × 10^3^ (*p* < 0.001)
IISM3	0.0974	0.0013	0.0935	0.1001	406.8 (*p* < 0.001
IISM4	0.0220	0.0012	0.0204	0.0286	89.4 (*p* < 0.001)
IISM5	0.0280	0.0010	0.0259	0.0329	126.5 (*p* < 0.001)

**Table 5:** Subject cumulative deviation for only 7th and 8th frequency bins (high frequencies, CD_78_) analysis by inter-pulse interval sequence model (IISM) groups.

	Number of Subjects Exceeding CD_78_ Thresholds (Frequency Bins 7 + 8)			
	IISM1 CD_78_ Mean + 1 S.D.	IISM1 CD_78_ Mean + 2 S.D.	IISM1 CD_78_ Mean + 3 S.D.	IISM1 CD_78_ Mean + 4 S.D.
IISM1	7 (13.5%)	4 (7.7%)	1 (1.9%)	0 (0.0%)
IISM2	52 (100%)	52 (100%)	52 (100%)	52 (100%)
IISM3	52 (100%)	52 (100%)	52 (100%)	52 (100%)
IISM4	52 (100%)	52 (100%)	52 (100%)	52 (100%)
IISM5	52 (100%)	52 (100%)	52 (100%)	52 (100%)

## Data Availability

The original contributions presented in this study are included in the article/Supplementary Material. Further inquiries can be directed to the corresponding author.

## Supplementary Materials

The following supporting information can be downloaded at: https://www.mdpi.com/article/10.3390/bioengineering13030279/s1, File S1: Python code; Video S1: light breakbeam mechanism and coding display in action.

## References

[1] World Health Organization (2022). Global Report on Infection Prevention and Control.

[2] Adrie C., Garrouste-Orgeas M., Ibn Essaied W., Schwebel C., Darmon M., Mourvillier B., Ruckly S., Dumenil A.-S., Kallel H., Argaud L. (2017). Attributable mortality of ICU-acquired bloodstream infections: Impact of the source, causative micro-organism, resistance profile and antimicrobial therapy. J. Infect..

[3] Stewardson A.J., Marimuthu K., Sengupta S., Allignol A., El-Bouseary M., Carvalho M.J., Hassan B., Delgado-Ramirez M.A., Arora A., Bagga R. (2019). Effect of carbapenem resistance on outcomes of bloodstream infection caused by Enterobacteriaceae in low-income and middle-income countries (PANORAMA): A multinational prospective cohort study. Lancet Infect. Dis..

[4] World Health Organization (2024). Guidelines for the Prevention of Bloodstream Infections and Other Infections Associated with the Use of Intravascular Catheters: Part 1: Peripheral Catheters.

[5] Zingg W., Pittet D. (2009). Peripheral venous catheters: An under-evaluated problem. Int. J. Antimicrob. Agents.

[6] Murray C.J.L., Ikuta K.S., Sharara F., Swetschinski L., Robles Aguilar G., Gray A., Han C., Bisignano C., Rao P., Wool E. (2022). Global burden of bacterial antimicrobial resistance in 2019: A systematic analysis. Lancet.

[7] Rosenthal V.D., Maki D.G., Jamulitrat S., Medeiros E.A., Todi S.K., Gomez D.Y., Leblebicioglu H., Abu Khader I., Novales M.G.M., Berba R. (2010). International Nosocomial Infection Control Consortium (INICC) report, data summary for 2003-2008, issued June 2009. Am. J. Infect. Control..

[8] Kamada S., Mosier R., El-Khalili T., Triantis S., Yang R. (2023). Scoping Review of Early Intravenous Infiltration and Extravasation Detection Devices. J. Infus. Nurs..

[9] Hirata I., Mazzotta A., Makvandi P., Cesini I., Brioschi C., Ferraris A., Mattoli V. (2023). Sensing Technologies for Extravasation Detection: A Review. ACS Sens..

[10] Brown S.J., Warner J.B. (1980). Measuring Security Price Performance. J. Financ. Econ..

[11] Vaidyanathan P.P. (2024). Signals, Systems, and Signal Processing.

[12] Mukherjee S., Bassler B.L. (2019). Bacterial quorum sensing in complex and dynamically changing environments. Nat. Rev. Microbiol..

[13] Guerreiro H., Wortmann N., Andersek T., Ngo T.N., Frölich A.M., Krause D., Fiehler J., Kyselyova A.A., Flottmann F. (2022). Novel synthetic clot analogs for in-vitro stroke modelling. PLoS ONE.

[14] Ruiz-Giardin J.M., Chamorro I.O., Ríos L.V., Aroca J.J., Arata M.I.G., López J.V.S., Santillán M.G. (2019). Blood stream infections associated with central and peripheral venous catheters. BMC Infect. Dis..

[15] Brown A.E., Litvinov R.I., Discher D.E., Purohit P.K., Weisel J.W. (2009). Multiscale mechanics of fibrin polymer: Gel stretching with protein unfolding and loss of water. Science.

[16] Pichon A., Roulaud M., Antoine-Jonville S., de Bisschop C., Denjean A. (2006). Spectral analysis of heart rate variability: Interchangeability between autoregressive analysis and fast Fourier transform. J. Electrocardiol..

[17] Guillery R.W., Sherman S.M. (2011). Branched thalamic afferents: What are the messages that they relay to the cortex?. Brain Res. Rev..

